# Development and Optimisation of Novel Polymeric Compositions for Sustained Release Theophylline Caplets (PrintCap) via FDM 3D Printing

**DOI:** 10.3390/polym12010027

**Published:** 2019-12-21

**Authors:** Deck Khong Tan, Mohammed Maniruzzaman, Ali Nokhodchi

**Affiliations:** 1 Pharmaceutics Research Laboratory, School of Life Sciences, University of Sussex, Brighton BN1 9Q, UK; D.Tan@sussex.ac.uk; 2 Pharmaceutical Engineering and 3D Printing (PharmE3D) Lab, Division of Molecular Pharmaceutics and Drug Delivery, College of Pharmacy, University of Texas at Austin, Austin, TX 78712, USA; 3 Drug Applied Research Center and Faculty of Pharmacy, Tabriz University of Medical, Sciences, Tabriz 51664, Iran

**Keywords:** 3D printing, HME, sustained release, PrintCap, polymeric carriers, drug delivery

## Abstract

This study reports a thorough investigation combining hot-melt extrusion technology (HME) and a low-cost fused deposition modelling (FDM) 3D printer as a continuous fabrication process for a sustained release drug delivery system. The successful implementation of such an approach presented herein allows local hospitals to manufacture their own medical and pharmaceutical products on-site according to their patients’ needs. This will help save time from waiting for suitable products to be manufactured off-site or using traditional manufacturing processes. The filaments were produced by optimising various compositions of pharmaceutical-grade polymers, such as hydroxypropyl cellulose (HPC), Eudragit^®^ (RL PO), and polyethylene glycol (PEG), whereas theophylline was used as a model thermally stable drug. For the purpose of the study, twin-screw hot-melt extrusion (HME) was implemented from the view that it would result in the formation of solid dispersion of drug in the polymeric carrier matrices by means of high shear mixing inside the heated barrel. Four filament compositions consisting of different ratios of polymers were produced and their properties were assessed. The mechanical characterisation of the filaments revealed quite robust properties of the filaments suitable for FDM 3D printing of caplets (PrintCap), whereas the solid-state analyses conducted via DSC and XRD showed amorphous nature of the crystalline drug dispersed in the polymeric matrices. Moreover, the surface analysis conducted via SEM showed a smooth surface of the produced filaments as well as caplets where no drug crystals were visible. The in vitro drug release study showed a sustained release profile over 10 h where about 80% of the drug was released from the printed dosage forms. This indicates that our optimised 3D printed caplets could be suitable for the development of sustained release on-demand drug delivery systems.

## 1. Introduction

Since the invention of additive manufacturing, many industries have been able to adopt this technology due to the numerous benefits it could bring. One of the most attractive features of 3D printing is its ability to produce objects of any shape in a cost-effective way at any time [1]. For this reason, 3D printing has a large potential in the use of pharmaceutical and biomedical applications. Due to the current rise in the development of personalised medicines, there is a need for a cost-effective method to produce these patient-tailored medicines in a short time frame after diagnosis [2]. In theory, 3D printing should be able to produce these medicines, in particular, the solid dosage forms that are adjusted according to the patient’s need [3]. The use of 3D printing in producing medicines also allows hospitals and pharmacies to manufacture the medications for patients immediately after consultation. This is particularly beneficial for hospitals in remote areas so that they can supply their own patients with just-in-time medications. Apart from that, 3D printing can be used to produce dosage forms with complex shapes and different release patterns cost-effectively, which can otherwise be relatively expensive and difficult to achieve with traditional manufacturing methods [4,5,6]. It is quite difficult to produce products with high complexity via traditional pharmaceutical processes such as compression and encapsulation, and it is not effective in terms of producing personalised products [7]. With 3D printing, it is possible to fabricate medicines of different shapes, sizes, and structures to tailor the release patterns of the drug [8]. It is also more cost-effective to produce personalised medicines through 3D printing as the designs of the medicine can be easily optimised using a computer. Besides, personalised medicines are often only required in small batches, which makes the 3D printing technique economically feasible. Three-dimensional printing is also useful for on-demand manufacturing right at the point of need, where medicine is required immediately in critical situations. The ability to produce medicines only when they are required is particularly useful for drugs with low-stability. This means the maximum efficiency of the drug can be obtained as the storage time is minimised and hence degradation can also be minimised [9]. Since the U.S. Food and Drug Administration’s (FDA) approval of the first 3D printed drug, Spritram^®^, there has been plenty of research that involves the 3D printing of various types of drug delivery systems [10,11,12]. The most easily accessible and low-cost 3D printing is fused deposition modelling (FDM). There has been much research on the polymeric filaments for FDM 3D printers that can be used in biomedical applications. This is because there are no commercially available drug-loaded filaments for medical applications [13]. The research on drug-loaded 3D printed filaments can help discover cost-effective ways to produce personalised drug delivery systems. The 3D printing technology as a fabrication method for personalised drug delivery system seems promising. Much of the works undertaken were in research labs and were successful in translating into the commercial scale. Liang et al. reported the first human study for a 3D printed mouthguard as a drug delivery device [14]. The authors utilised various compositions of polylactic acid (PLA) or polyvinyl alcohol (PVA) to develop a tailored oral device for tuneable sustained release drug delivery of anti-inflammatory corticosteroid and were fabricated using hot-melt extrusion (HME) technology. The authors found that all developed devices showed a sustained release pattern of the drug over 14 days, controlled by the nature of the polymer used instead of the actual printing parameters such as shape, print-fill, etc.

In this study, HME was utilised to develop drug-loaded polymeric filaments for their potential application in FDM 3D printing. HME is a well-established technique that has been used for pharmaceutical applications [15,16,17,18]. Many studies have also proven that HME can produce API-loaded filaments for FDM 3D printing [19,20,21]. However, the main concern of the filaments produced is mechanical resilience and their rheological properties, which are the main factors that determine its printability. Therefore, this study involves the development and optimisation of a pharmaceutical-grade filament that has suitable mechanical and rheological properties and can be used for FDM 3D printing. During FDM 3D printing, the filament is fed into a heated nozzle head for the melting of the filament through a motorised gear. The gear exerts stress and strain onto the filament during the feeding process. Therefore, the filaments should be rigid and stiff enough longitudinally. It should also have enough plasticity so that it can withstand the stress exerted transversely. The mechanical resilience of the filament is dependent on the type of polymers used as a drug carrier. 

This study combines HME and FDM 3D printing in order to achieve a cost-effective fabrication platform for a patient-tailored drug delivery system. This continuous manufacturing platform has been previously proven feasible by Zhang et al. to produce controlled release tablets [19]. Most of the reported studies in the literature have utilised HME as an efficient method to produce drug-loaded filaments with the most immediate potential for the unit dose fabrication and to meet the needs of patient-specific doses [13,21,22]. However, the work reported here involves the development of a type of biocompatible filament by mixing several pharmaceutical-grade polymers, plasticiser, and an API through the HME technique. The polymers are hydroxypropyl cellulose (HPC), Eudragit^®^ RL PO, and polyethylene glycol (PEG). The model drug-loaded is theophylline, which is widely known as a thermostable drug [13]. The study shows that different release patterns can be achieved by changing the concentration of HPC and Eudragit. This composition of polymers would be suitable for a wide range of modified release drug delivery systems and can be easily tailored according to the patient’s need via an economically viable manufacturing platform (only a little change in polymeric content is required to tailor the release pattern). 

In this study, the properties and the printability of the extruded filaments were compared. The filament produced needs to have good flexibility and ductility so that it can be fed into the printing head of the FDM 3D printer. HPC is a derivative of cellulose and is a type of thermoplastic with high amorphous content. As a result, it has high molecular mobility and plasticity [23]. Due to its physical properties, HPC has been chosen to be made into FDM printable filament as it provides ductility to the filament [24,25]. HPC has been widely used in the pharmaceutical industry as a tablet binder, thickening agent, viscosity increasing agent, and coating agent [23]. A study carried out by Sarode et al. shows that HPC can be used to produce chemically stable solid dispersions of poorly and highly water-soluble drugs [26]. The polymer HPC can effectively convert the crystalline drug to amorphous form, improving the dissolution and making the drug a sustained release type. Traditionally, HPC has been used to produce extended release tablets [27]. However, HPC is hygroscopic and has the tendency to absorb moisture from the atmosphere. Its typical equilibrium moisture content value at 25 °C is 4% *w/w* at 50% relative humidity [23]. Therefore, suitable storage conditions with low humidity are required for the products. Eudragit^®^ RL PO is an amorphous polymer and is an ammonio methacrylate copolymer type A. It has been used for sustained release products [28]. It is highly permeable to water but is not water-soluble. According to the Heckel analysis by Dave et al., the deformation behaviour of Eudragit^®^ RL PO is similar to a typical plastic material [29]. The presence of Eudragit can impart flexibility to the filament, which is essential for the use in FDM 3D printing. A study carried out by Kotiyan shows that Eudragit^®^ RL PO can be used to prevent crystallisation [30]. This would be useful in keeping the drug-loaded polymer matrix in amorphous form [31]. PEG is a semi-crystalline polymer that has been used in a wide variety of pharmaceutical formulations for controlled release systems as it can prolong disintegration of a dosage form [32]. It is biocompatible, stable, and hydrophilic. Its hydrophilicity can improve aqueous solubility and bioavailability of solid dispersion. PEG has also been effectively used as a plasticiser in polymer matrices [33,34,35]. By adding Eudragit and PEG into the formulation, the flexibility and plasticity of HPC filament can be improved so that it can be used for 3D printing. Korte et al. also reported that PEG is a suitable plasticizer for producing 3D-printable filaments [13]. The model drug, theophylline, is an odourless white crystalline powder with a bitter taste. Theophylline is chosen as it has a high melting point of 273 °C, which appears as an ideal candidate to withstand thermal processing. Due to its thermal stability at high temperature, it is suitable to be used both in HME and FDM 3D printing. However, theophylline is only sparingly soluble in water and alcohol. The solubility of theophylline in water is 5.5 mg/mL, whereas in methanol and ethanol is 0.699 mg/mL and 15.19 mg/mL, respectively [36,37]. Due to the advantages of HPC and PEG in solid dispersions and their optimum thermoplastic properties for thermal extrusion, these polymers were chosen for the development of sustained release products by means of HME [38,39,40]. The shear mixing and kneading during the extrusion process allows homogenous mixing of the polymers and the drug as the drug is molecularly dispersed in the polymer matrix. 

Therefore, the aim of this study was to develop and optimise various novel polymeric compositions for FDM 3D printing of sustained release drug delivery systems by means of a twin-screw extruder. The novel polymeric composition can achieve various release patterns by just altering the percentage of HPC and Eudragit. This type of composition can be used to produce a wide range of modified release drug delivery systems cost-effectively. Previous studies have successfully used HPC to produce drug-loaded filaments for the 3D printing of tablets [11,41,42]. However, HPC itself is hygroscopic, causing HPC-based filaments to have very short usage life [43]. The HPC filament can absorb moisture from the air easily, causing it to be very brittle. Therefore, it cannot be used for 3D printing alone. Hence, it is mixed with Eudragit and PEG to improve its flexibility. The polymeric filament produced would be suitable to load other types of low-solubility APIs for their improved bioavailability in other medical applications. However, thermolabile APIs are not suitable as the printing temperature is high (195 °C), which could lead to degradation. The developed formulation can be used to 3D print specific shaped tablets and implants in various applications such as paediatric applications [44] as well as in low-resource settings. The printability of the filaments was assessed thoroughly by outlining the scope of 3D printing of a caplet out of these filaments. The mechano-chemical properties as well as in vitro drug release of the filaments and the 3D printed caplets were studied.

## 2. Materials and Methods

### 2.1. Materials

Nisso Hydroxy Propyl Cellulose (HPC) (Grade SSL) from Nippon Soda Co., LTD. (Tokyo, Japan) was used. Eudragit^®^ RL PO powder used was from Evonik Industries (Darmstadt, Germany). Poly (ethylene glycol) (PEG), average M.W. 6000, was purchased from Fisher Scientific (Loughborough, UK). Theophylline 99+% anhydrous purchased from Fisher Scientific (Loughborough, UK) was used as a model drug with a high melting point of around 273 °C. All materials were used as received.

### 2.2. Preparation of Theophylline-Loaded Filaments

The bulk HPC, Eudragit^®^ RL PO, PEG, and the drug were used and mixed together in a pestle and mortar. The mixed powder was then transferred into a bottle and further mixing was carried out using a Turbula shaker mixer (Eskens, Alphen aan den Rijn, Netherlands) for 15 min. The ratios of HPC:Eudragit:PEG:theophylline used to develop filaments are shown in Table 1. The mixed powder was then used to produce filaments via a customised twin-screw extruder L/D 20 (assembled by Point1Controls/R Controls, Stoke-on-Trent, UK) at a screw speed of 60 rpm. The temperature profiles for the 4 zones (Z1:Z2:Z3:Z4) were kept at 70 °C:100 °C:110 °C:110 °C, and the die at 110 °C. The heat soak time of the extruder was set to 5 min. The filaments extruded were collected using a filament winder and the diameters of the filaments were measured using Vernier calliper as they existed in the extruder die. Only filaments with a diameter of below 1.75 mm were used for 3D printing as the size of the 3D printer nozzle for feeding the filament was 1.75 mm. 

### 2.3. Design and Fabrication of Tablet

**Figure 1:**
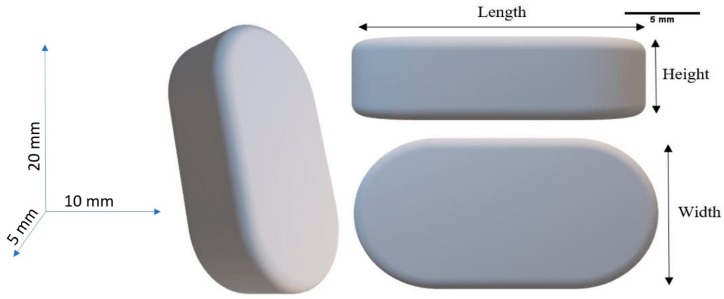
Three-dimensional CAD model of the tablet for 3D printing.

The extruded filaments were printed into tablets (caplets) using the Makerbot Replicator 2X (Makerbot Inc., NY, USA). The CAD model of the tablet was designed using Solidworks (Dassault Systemes, Waltham, MA, USA). Figure 1 shows a computer-generated 3D model of the tablet used for 3D printing. The 3D model was then saved as an STL file and imported to Makerbot Desktop software (Makerbot Inc., NY, USA) for slicing. The tablet had a height of 5 mm, width of 10 mm, and length of 20 mm. The layer height for printing was set to 150 µm. The printing temperature and bed temperatures were set at 195 °C and 60 °C, respectively, and the print infill was set to 100%. 

### 2.4. Differential Scanning Calorimetry (DSC)

A DSC 4000 system (Perkin Elmer, Waltham, MA, USA) was used to study the glass transition temperature and the melting temperature of all the individual substances, the powder mixture, the extruded filaments, and the 3D printed tablets. The samples used for each of the DSC scans were about 5 mg. The samples were placed in an aluminium pan, and an additional empty aluminium pan was used as a reference. Nitrogen gas was used as the purged gas at a flow rate of 20 mL/min in all the DSC experiments. All samples were heated from room temperature to 300 °C at a heat rate of 10 °C/min. The data were collected and analysed with Pyris software (Perkin Elmer, Waltham, MA, USA). 

### 2.5. X-Ray Powder Diffraction 

The crystallinity of the powder mixture, each individual powder, filament, and 3D printed tablet was assessed using a Siemens D500 X-ray Diffractometer (Siemens, Germany). The samples were scanned between 2 theta (θ) = 5° to 50° using 0.01° step width and 1 s time count. The divergence slit was 1 mm and the scatter slit was 0.6 mm. The X-ray wavelength was 0.154 nm in the Cu source and at a voltage of 40 kV.

### 2.6. Mechanical Characterisation (Texture Analyser)

Tensile testing was carried out on the filaments using TA.XT Plus Texture Analyser (Stable Micro Systems, Godalming, UK). A load cell of 50 kg was used and a tensile grip probe set was used to measure the tensile strength of the filaments. The filaments were cut into thin cylinders of 10 cm length. The test was set up as a tension test with a maximum applied force of 50 kg. The test stopped as soon as the filament broke. The data were collected and analysed using the Exponent Software version 7.0.3.0 (Stable Micro Systems, Godalming, UK).

### 2.7. Scanning Electron Microscope (SEM)

SEM was carried out using the JEOL JMS 820 (Freising, Munich, Germany) to study the morphology of the filaments and the printed samples. The samples were sputter-coated with gold under vacuum conditions using the Edwards S-150 sputter coater (Edwards High Vacuum Co. International, Albany, NY, US) for electrical conductivity. The accelerating voltage was at 3 kV at various magnifications. 

### 2.8. In Vitro Drug Release Study

An in vitro dissolution test was carried out on three randomly selected tablets from each formulation for drug release study. The mass of the samples was first weighed before dissolution and then weighed again after the dissolution test when the samples were dried. The dissolution parameters were chosen according to the US Pharmacopoeia (USP) 36 monograph ‘Theophylline Extended-Release Capsules Test 6′. Determination of the in vitro drug release was performed using USP type I dissolution apparatus (708-DS Dissolution Apparatus, Agilent Technologies, Santa Clara, CA, USA) in 1000 mL of 0.05 M phosphate buffer solution (pH 6.6) at 37 ± 0.3 °C with a rotation speed of 100 rpm. The drug concentration of the dissolution medium was measured using a Cary 60 UV-Vis Spectrophotometer (Agilent Technologies, Santa Clara, CA, USA) at a wavelength of 271 nm in a 1 cm cell versus a blank solution consisting of a phosphate buffer (pH 6.6). The samples were drawn automatically using an Agilent 810 peristaltic pump (Agilent Technologies, Santa Clara, CA, USA) and sampling was done every 5 min for the first hour, every 20 min of the following 2 h, and every 60 min for the last 10 h. The release profiles were plotted as percentages of cumulative drug release versus time.

## 3. Results and Discussions

### 3.1. Thermal Analysis

DSC is a highly sensitive technique in studying the thermal transition of materials as a function of heat-flow. In the HME process, the powder mixture was thermally stressed. Hence, it is important to study the changes in the materials when there is a change in temperature. It has been proven that theophylline has good thermal stability and can be processed using HME [13,45,46]. However, DSC measurements have been carried out on the pure substances, the powder mixture, the extrudates, and the 3D printed products to study the solid state of the drug in the formulations, as well as to ensure its thermal stability and miscibility. 

**Figure 2:**
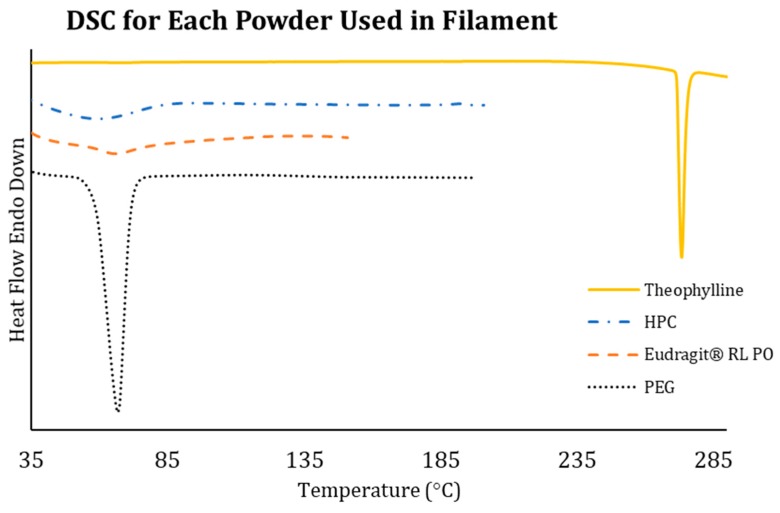
DSC thermal transitions of the bulk materials (theophylline, HPC, Eudragit^®^ RL PO, and PEG).

Figure 2 shows the DSC measurements for each individual bulk material (theophylline, HPC, Eudragit^®^ RL PO, and PEG) before mixing. Theophylline is a crystalline drug and has a sharp endothermic peak, which is the melting point at around 273 °C. The DSC result of theophylline shows that the ΔH value of theophylline is around 68.14 J/g. This shows that theophylline would be a suitable model drug for HME and FDM 3D printing processes as these processes require a high temperature. The study also shows that HPC and Eudragit^®^ RL PO have high amorphous content as the DSC curves for both materials do not show obvious endothermic events. However, the amorphous materials were expected to exhibit a thermal transition by means of a step change, which represents the glass transition temperature (*T*_g_). The *T*_g_ is the temperature when the molecules in the material start to transition from a glassy state to a soft, rubbery state [47]. This is the temperature when the materials soften. According to the technical data sheet of the HPC Grade SSL from the manufacturer, it exhibits two softening temperatures: one at 75 °C and the second at 183 °C [48]. From the DSC curve, the *T*_g_ of HPC was found to be around 76 °C. The dip before the slope of the glass transition could be caused by the plasticising effect due to the presence of moisture in the HPC polymer. The *T*_g_ for Eudragit was around 66 °C, which is similar to the literature [31,49]. PEG is a semi-crystalline polymer and the DSC showed a melting peak at around 67 °C. The calculated heat enthalpy value (ΔH) of PEG is approximately 252.7 J/g. 

**Figure 3:**
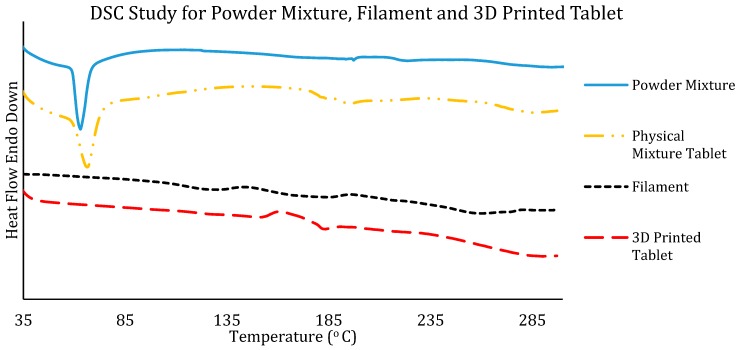
DSC results obtained for the powder mixture (physical blends of the bulk drug and polymers), the physical mixture for tablet (tablet formulation using the bulk materials for direct compression for comparison with the 3D printed tablets), HME extruded filaments, and the FDM 3D printed tablet.

The DSC curve for the powder mixture in Figure 3 mimics the temperature cycle the powder mixture went through during the extrusion process. This is to ensure the thermal behaviour of the powder mixture as it is required to go through the processes of high temperature. The powder mixture of HPC, Eudragit, PEG, and theophylline was placed in the sample pan for heating. However, the results obtained only reflect the true thermal events, i.e., thermal transition by means of dynamic heat-flow experienced by the samples studied. In reality, during the extrusion process, the mixture underwent kneading and shear mixing in addition to the thermal processing at 110 °C, which is not reflected in the DSC result. The extruded filament was then fed into a hot printing head at a temperature of 195 °C (this high temperature was used to avoid any clog in the printing head without any degradation of the drug). During the printing process optimisation step, it was found that in order to properly soften the polymeric filament such that it can be dispensed from the printing head of the FDM printer, the optimal printing temperature required is 195 °C. In a lower processing temperature, the filaments tend to clog the printing head owing to less fluidity. Therefore, the heating of the powder mixture in the DSC study was carried out up to 300 °C. The DSC curve only showed an endothermic peak at around 60 °C for the heating of the powder mixture. This is the melting point of PEG and confirms the presence of PEG in the powder mixture. The DSC curve of the physical mixture tablet shows a similar pattern to the powder mixture. This is because the mixture has not been thermally processed through HME.

The DSC curves in Figure 3 also contain the DSC study for the HME-produced theophylline-loaded filament. The DSC curve shown here is for the formulation of F2 filaments. From the DSC results for the filament, we can see that the peaks for the PEG and theophylline melting points have been depressed. There were no obvious endothermic peaks on the DSC curve due to the lack of crystallinity in the filament. This has proven that a complete amorphous system has been developed through the process of twin-screw extrusion. It has also been reported that HPC can effectively convert the crystalline drug to amorphous form [23], which is also shown in the DSC results obtained. The DSC results for the filament showed a very small step change at around 160 °C, which could be due to the softening of the mixture or the enthalpy relaxation of the new amorphous filament. The DSC results of the 3D printed tablet exhibited amorphous content as there was no occurrence of endothermic peaks during the heating cycle. There is a small step change at around 170 °C, which could be the *T*_g_ of the 3D printed tablet. The high temperature of 3D printing could have slightly shifted the *T*_g_ of the tablet from the filament. Like the filament, the thermal transition corresponding to the presence of crystalline theophylline could not be seen, which further confirmed that the process of HME converted the crystalline theophylline to amorphous. The DSC study shows that the mixture is thermally stable to be used in HME at 110 °C, and the filaments produced are suitable for FDM 3D printing at a temperature of 195 °C. The extruded filament and the 3D printed tablet also have thermal stability of up to 300 °C (the evidence of thermal degradation observed in DSC).

### 3.2. X-Ray Powder Diffraction (XRPD)

**Figure 4:**
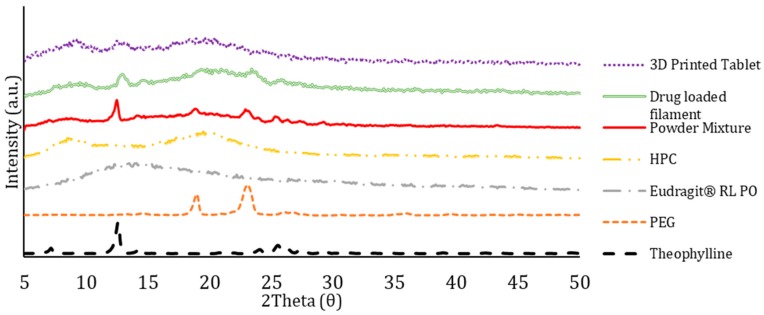
XRPD patterns of the bulk polymers and drug, powder mixture, filament, and 3D printed tablet.

X-ray powder diffraction has always been used for qualitative analysis to determine the crystallinity of a material. XRPD study was carried out on all the individual bulk powders, the powder mixture, the extruded filament, and the 3D printed tablet. The results from the XRPD for formulation F2 are shown in Figure 4. The scans from theophylline and PEG showed obvious crystalline peaks, which means these two materials have high crystallinity in their bulk forms. The diffractograms for HPC and Eudragit^®^ RL PO confirmed that these two powders are highly amorphous, reflected by no obvious peaks. The powder blend showed some crystallinity as expected as the powders were only physically mixed at room temperature. The crystallinity in the powder mixture was due to the presence of crystalline theophylline and PEG. As the powders were physically mixed, there is not much reaction between the powders that could highly affect the crystallinity of the mixture. However, the crystallinity is slightly lower in the powder mixture than the pure theophylline and PEG individually. This is expected due to the presence of amorphous polymers (Eudragit and HPC) in the formulations. The amorphous polymers would help reduce the crystallinity through embedding the crystalline drug into its matrices during the mixing. As a result, the drug entrapped within the amorphous polymer chains would exhibit reduced crystallinity. As for the theophylline-loaded filament, the result showed that the filament was highly amorphous with a very small amount of crystallinity reflected by the presence of low intensity peaks. A quite similar phenomenon was observed on the 3D printed tablets. The peaks that can be seen due to the presence of PEG and theophylline are not obvious in the 3D printed tablet. This has proven that the process of twin-screw extrusion successfully converted the crystalline theophylline into amorphous form by molecular dispersion of the drug molecules into the polymer matrix. These results are in line with the studies from the DSC of the filament and 3D printed tablets, which further confirms that the model drug, theophylline, converted to its amorphous form as a function of being molecularly dispersed into the polymer matrix.

### 3.3. Mechanical Properties

**Figure 5:**
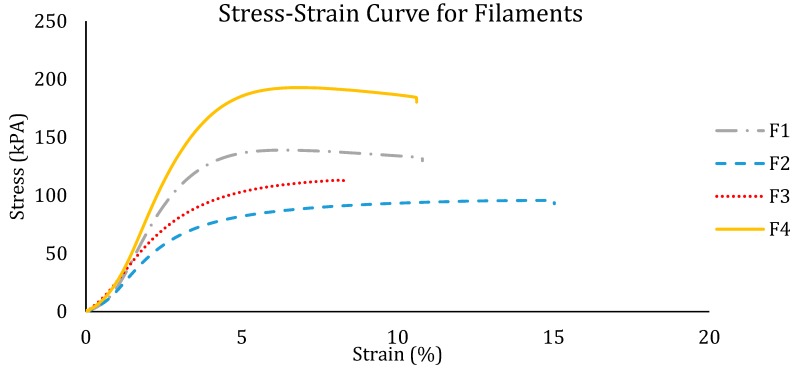
Stress-strain curve from the tensile testing of all theophylline-loaded filaments.

The mechanical strength of the extruded filament is very important as the FDM printing process exerts a considerable amount of force onto the filament. The filaments must have the right balance between ductility and stiffness for them to be printable. In the FDM 3D printing process, a gear is used to feed the filament into the printing head. The gear exerts shearing and tensile stresses onto the filament. Therefore, the filament must be ductile and strong enough to withstand the transversally applied pressure. Tensile testing was carried out to determine the mechanical resilience of the extruded filaments. Figure 5 shows the stress-strain curve plotted from the results obtained through the tensile testing of the four different filaments. The strength of the filaments can be calculated through the stress-strain curve. Yield strength is the minimum stress under which the material deforms permanently (i.e., the stress at which the material stops behaving elastically is called the yield strength). This is the highest point where the strain increases proportionally with stress. Ultimate tensile strength is the maximum stress that a material can withstand while being stretched before breaking. This is the highest point of the stress-strain curve. Young’s modulus measures the stiffness of the material. It describes the elastic properties of the filament undergoing tension in one direction. This can be obtained by calculating the gradient of the proportional section on the curve. The calculated results for the yield strength, ultimate tensile strength, Young’s modulus, and elongation at break are in Table 2. The results show that F1 and F4 have a similar strain. F2 has the highest strain, which means it can be stretched the most before failing. From the curve in Figure 5, F4 has the highest stress values, which means it can withstand the most force. This could be due to the high content of HPC as the high percentage of HPC in the composition makes the filament more ductile. However, the second-highest yield strength is F1, which has the highest concentration of Eudragit. The presence of Eudragit imparts plasticity to the filament, causing it to have a high yield strength. This study shows that both HPC and Eudragit can provide the strength of the filaments so that they are suitable for 3D printing. The percentage of HPC and Eudragit can be easily changed for different purposes but still be able to produce filaments with adequate strength for 3D printing. The results from tensile testing can only be used as a reference as it only represents the uniaxial force that the filaments can withstand.

### 3.4. 3D Printing Process 

**Figure 6:**
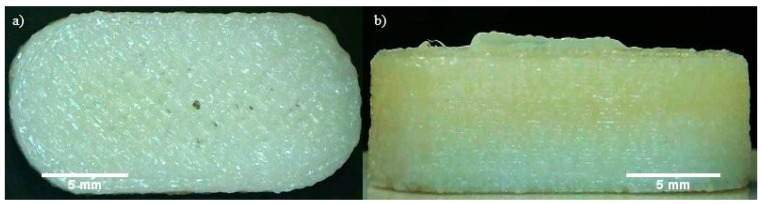
3D printed tablet from the filament F2: (**a**) top view; (**b**) side view.

**Figure 7:**
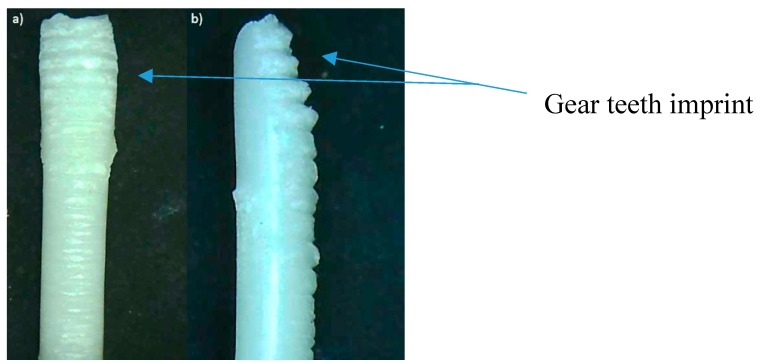
Failed filament during FDM 3D printing: (**a**) top view; (**b**) side view.

The theophylline-loaded filaments were used to 3D print tablets using a Makerbot Replicator 2X FDM 3D printer. The temperature of printing was set at 195 °C, which is very close to the normal printing temperature of PLA filaments (around 190–230 °C). Despite the filament not being as strong as the commercial filaments available, as proven from the tensile testing, the filament can still withstand the shear forces and stress experienced through the extruder of the FDM 3D printer. The printing of a tablet took approximately 10 min including the heating up of the printing nozzle and printing bed. Once the printer is heated up, the printing time required for one tablet is only 5 min. Figure 6 shows an example of a 3D printed tablet produced using the filament of formulation F2. All four filaments were printable. F1, F2, and F3 filaments could produce good quality 3D printed tablets. Only the tablets 3D printed using F4 filaments have poor quality. The quality of the tablets was assessed by visual inspection on the shape and the surface finish of the printed tablet. The quality of printing was assessed by the difficulty in removing the printed tablet from the printing bed after printing without damaging the tablet. Tablets printed from F1 and F2 filaments can be removed easily from the printing bed. They also have a smooth surface finish. The surface finish of F3 tablets was not as smooth and the tablets were more brittle, causing some difficulties in removal from the printing bed. However, the F3 tablets can still be removed from the bed without damaging them with extra care and attention. F4 printed tablets had good adhesion to the printing bed, causing them to be difficult to remove. Even after being removed by means of physical force, i.e., by tweezer, the tablets left some remnants on the print bed, which altered its original shape. As a result, the surface finish of the printed tablet was not as good as the other formulations. The tablets were brittle. Therefore, F4 is not ideal particularly for the fabrication of the tablet as the consistency of each tablet will be affected. The high content of HPC in F3 and F4 could have contributed to its brittleness. In order to achieve a good quality print, the filament needs to have enough plasticity and smoothness, which is provided by the presence of Eudragit. The higher the content of Eudragit, the higher the plasticity and flexibility of the filaments. However, the plasticity of the filament cannot be too high as it may lose its ductility, which is needed to withstand the forces exerted onto the filaments during printing. The filament that has too much plasticity could be easily deformed, which could be an issue during the feeding process of FDM 3D printing. This is because the teeth of the gear may leave an imprint onto the filament and change the shape of the filament, which is shown in Figure 7. The filament that has high flexibility is also difficult to keep its shape and remain straight, which makes it difficult to be fed into the small hole of the printing head. However, high plasticity allows a smoother surface finish as the melted filament has a higher viscosity, making the extrusion during 3D printing easier. The printability of all four filaments is shown in Table S1. 

Extra attention was required for the storage condition of the filaments extruded. This is because the filament can absorb moisture from the environment like many other commercially available filaments such as the PLA filaments. HPC has a high affinity to water molecules. If the filament is left in the air for a long period of time, such as overnight at above 55% RH, the filament will turn hard and brittle due to the absorption of moisture. A brittle filament will fail at the gear during the feeding of the filament into the printing nozzle. The brittle filament will be crushed by the force exerted by the feeding gear. Therefore, it is recommended that the filament should be used immediately for printing after being produced using the twin-screw extruder. Otherwise, filaments need to be stored in a dry environment to preserve and prolong the life of the filament. For this reason, the filaments were either used as extruded or kept in a desiccator in a sealed bag when not used in this study. It has also been noted that the filaments should always be stored in low humidity conditions (i.e., <45% RH) owing to the presence of hydrophilic excipients in the formulations. The filaments were all used for 3D printing within two days after the extrusion process. The filaments produced here were stored in a sealed bag with desiccant bags, which could reduce the humidity of the air in the sealed bag when not in use. A comparison of the texture of the stored filament with that of extruded fresh filaments did not show any significant differences in terms of the printing fidelity, texture, and physical properties of the formulations. 

### 3.5. Scanning Electron Microscopy (SEM)

**Figure 8:**
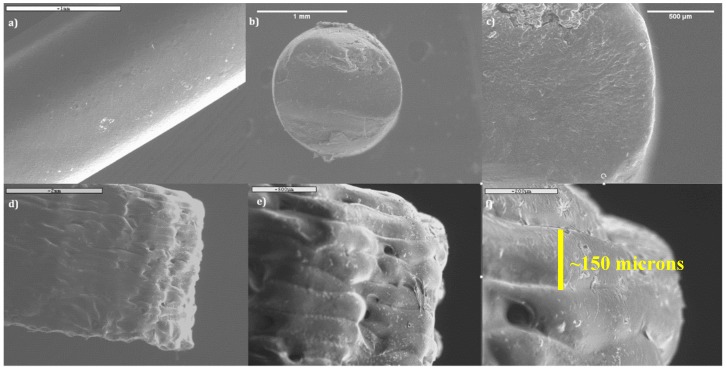
SEM images of theophylline-loaded filament (**a–c**) and 3D printed tablet (**d–f**). (**a**) Surface morphology of filament at magnification of 20×, (**b**) cross-section of filament at magnification of 20×, (**c**) cross-section of filament at magnification of 50×, (**d**) side view of 3D printed tablet at magnification of 20×, (**e**) side view of 3D printed tablet at magnification of 50×, and (**f**) side view of 3D printed tablet at magnification of 200×.

SEM images were obtained to study the morphology of the filament, cross-section, and the 3D printed tablets. The images of the filament surface and the filament cross-section are shown in Figure 8a–c. The SEM images showed that the filament has smooth surfaces and a very consistent round shape. There was no drug aggregation seen on the images, which further confirms the twin-screw extrusion has successfully converted the theophylline crystals into amorphous form because of its high intense mixing and torque generated during the process. SEM images of the 3D printed tablet are also shown in Figure 8d–f. For the 3D printed tablets, we only focused on the surface to assess the morphology and smoothness as well as printing accuracy as a function of the deposited layer thickness (~150 µm), in line with the overall aims of this paper. The SEM images clearly showed the multiple layers of the tablet that were deposited sequentially during the FDM 3D printing process. It can be seen from the images that the thickness of each layer was quite consistent. The thickness of each layer is 0.15 mm, which is the actual layer height adjusted during the printing process. This has proven that FDM 3D printing has high precision and accuracy. 

### 3.6. In Vitro Drug Release Study

**Figure 9:**
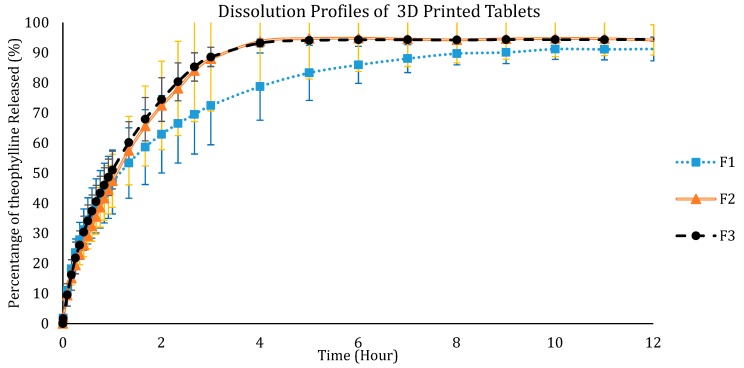
Dissolution profiles of the 3D printed tablets from filament formulations F1, F2, and F3.

**Figure 10:**
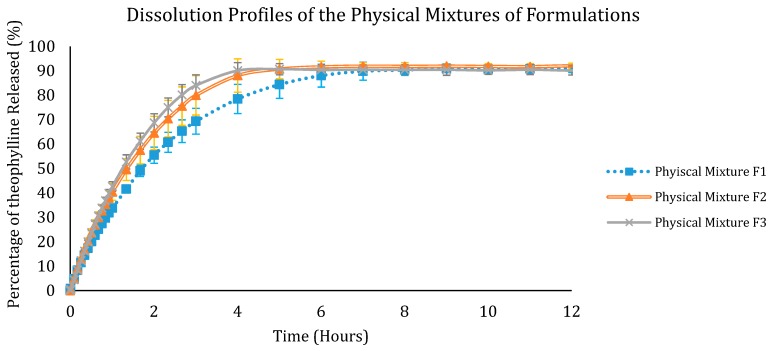
Dissolution profiles of the physical mixture tablets F1, F2, and F3 formulations.

After the development of theophylline-loaded filaments and the 3D printing of tablets, the drug loading of the filaments and the 3D printed tablets were determined. The samples were dissolved in ethanol and the drug concentration was measured using a Biochrom WPA Biowave II UV/Visible spectrophotometer (Biochrom Ltd., Cambourne, UK) at a wavelength of 271 nm. The percentage of theophylline in the filaments was 93.76 ± 2.76% and the drug content in the tablets was 92.46 ± 5.73%. This shows that there was no major degradation of the drug caused by thermal processing, i.e., HME and the FDM 3D printing. Dissolution studies were then carried out on the 3D printed tablets. All dissolution studies were carried out within two days after 3D printing of the tablets. The tablets were kept in low humidity conditions using a sealed bag with desiccants to reduce the amount of moisture absorbed by the tablets. The drug release profiles were shown in Figure 9 and Figure 10. Only F1, F2, and F3 tablets were studied as the 3D printed tablet of F4 did not show good quality. F1 tablets show the slowest release profile, and F3 the quickest release, showing that the higher the percentage of Eudragit, the slower the release. The time taken for theophylline to completely release from F1 tablets was around 10 h. The drug release in F2 and F3 tablets plateaued after 4 h. The drug release study shows that all three formulations are suitable for the purpose of sustained release delivery as the drug release continued for over 5 h. All the tablets showed drug release of more than 80%. For comparison, dissolution studies were also carried out for the physical mixtures for the three formulations. Figure 10 shows the dissolution profiles for the physical mixtures of the tablet formulations. Like the 3D printed tablets, F1 formulation has the slowest release whereas F3 has the fastest release; when compared the 3D printed tablets, they have almost similar or slightly faster release properties than the respective physical mixture. This could be due to the homogenous mixing of the powder mixture during the HME process resulting in the increase in amorphicity of the drug and thus slightly enhanced release rate. Nonetheless, all formulations showed sustained release over the experiment period (12 h).

All dissolution profiles for the 3D printed formulations were compared using the difference factor (*f1*) and the similarity factor (*f2*). The dissolution profiles can be considered similar when *f1* values are from 0 to 15 and *f2* values are from 50 to 100. The results from the comparison are shown in Table 3. The results show that all three formulations for the 3D printed tablets have equivalent dissolution profiles. 

**Figure 11:**
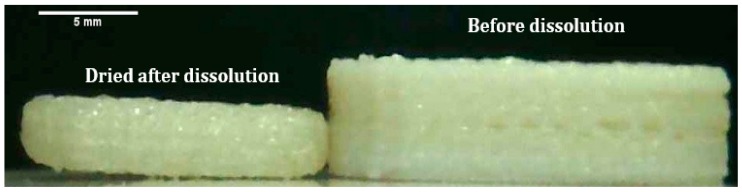
Tablet change in size after drying after dissolution study.

**Figure 12:**
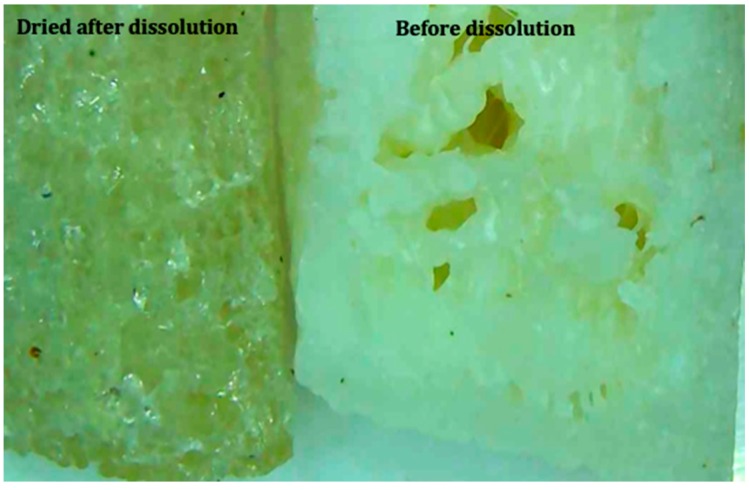
Cross-section image of tablet after (left) and before (right) dissolution.

The tablets for all the formulations did not dissolve completely during the dissolution studies and the original shape of the tablet was retained. This is because Eudragit was not soluble in the dissolution medium used and it managed to keep the shape of the tablet even after the drug was completely released. Figure 11 above shows that the tablet size changed after the tablet was dried after dissolution. The size of the tablet did not change when the tablet was removed from the dissolution medium as the dissolution medium was absorbed by the polymer, making the tablet swell. The two polymers used (HPC and PEG) are soluble in water but only one polymer, Eudragit, is not soluble. Eudragit is permeable to water, hence it is likely that Eudragit absorbed the dissolution medium. During the dissolution process, the HPC and PEG fully dissolved with the drug-loaded polymer. When the tablet was removed from the dissolution medium, the size and shape of the tablet were retained by the Eudragit. Once the medium absorbed by the Eudragit had completely evaporated, the shape of the tablet remained but it shrank in size. The tablets were weighed before and after the dissolution studies for the comparison of the change in mass. The changes were shown in Table 4. The mass of the dry tablet corresponds to the percentage of Eudragit present in the mixture. The tablets retained frame and shape after the drug release. Figure 12 shows the cross-section of the tablet after and before the dissolution. The white colour of the tablet was due to the presence of theophylline and HPC. Once the drug was released, the tablet became translucent. The image shows that the drug was completely released. 

This study shows that such polymeric composition could be useful for the development of a medical implant. This type of formulation is particularly useful to be used as a medical implant for drug delivery or monitoring the condition of the body. This is because the insoluble part of the system can still hold the sensing devices on the implant after the drug has completely been released.

## 4. Conclusions

This study showed that HME and FDM 3D printing, when combined together, can form a cost-effective and convenient process to produce sustained release drug delivery systems. The developed polymeric composition can be easily tailored through HME depending on the need for modified drug release patterns. This is particularly useful for the fabrication of personalised medicines and implants as it could save the waiting time of the patients. HME has been used to produce 3D printable filaments that can be loaded with thermally stable API. HME can also effectively convert a crystalline API into amorphous form, forming a solid dispersion. In order to produce FDM 3D printable filaments, the type of polymeric carrier chosen is essential. It must have suitable flexibility and ductility that can withstand the forces experienced during the FDM 3D printing process. In this study HPC (Grade SSL), Eudragit^®^ RL PO, and PEG mixed with theophylline as API produced 3D printable filaments and tablets thereof. All printed tablets showed sustained release profiles for over 10 h, which is suitable for sustained release drug delivery systems. 

## Figures and Tables

**Table 1:** The different ratios of (hydroxypropyl cellulose (HPC):Eudragit:PEG:theophylline) used for the fabrication of different filaments.

Formulation No.	Ratio of (HPC:Eudragit:PEG:theophylline)
F1	4:4:1:1
F2	5:3:1:1
F3	6:2:1:1
F4	7:1:1:1

**Table 2:** Calculated strength of the theophylline-loaded filaments.

Filament Formulation No.	Yield Strength (kPa)	Ultimate Tensile Strength (kPa)	Young’s Modulus (kPa)	Elongation at Break (%)
F1	110.3 ± 22.11	137.93 ± 18.24	43.21 ± 9.38	10.7 ± 3.73
F2	61.7 ± 1.59	95.7 ± 2.79	31.52 ± 2.64	15.1 ± 1.35
F3	68.9 ± 1.54	113.2 ± 5.91	31.49 ± 1.57	8.23 ± 1.05
F4	135.4 ± 7.91	192.8 ± 7.06	54.55 ± 3.72	10.6 ± 2.51

**Table 3:** Difference factor and similarity factor for dissolution profiles of 3D printed tablets.

Formulation No. Compared	Difference Factor (*f1*)	Similarity Factor (*f2*)
F1 and F2	12.63	55.99
F2 and F3	5.54	74.70
F3 and F1	12.73	55.18

**Table 4:** Change in mass of the dry tablets after dissolution studies.

Formulation No.	Tablet No.	Mass Before Dissolution (mg)	Mass of Dry Tablet After Dissolution (mg)
F1	1	629.6	280.9
	2	631.9	287.1
	3	653.2	286.4
F2	123	791.4	291.7
	2	784.0	272.3
	3	806.7	292.6
F3	123	633.3	194.6
	2	632.3	192.5
	3	625.5	190.8

## Supplementary Materials

The following are available online at https://www.mdpi.com/2073-4360/12/1/27/s1: Table S1: FDM printability and quality of all four theophylline-loaded filaments.
