# Supplementary material for: Development and Optimisation of Novel Polymeric Compositions for Sustained Release Theophylline Caplets (PrintCap) via FDM 3D Printing
Source: Polymers (Basel). 2019 Dec 21;12(1):27. doi: 10.3390/polym12010027 (PMC7023590; doi:10.3390/polym12010027)
Supplement: Supplementary file 1 [file polymers-12-00027-s001.pdf]

polymers

Article

Development and optimisation of novel polymeric compositions for sustained release theophylline caplets (PrintCap) via FDM 3D printing

Deck Khong Tan[1], Mohammed Maniruzzaman [2*] and Ali Nokhodchi[1*]

1 Pharmaceutics Research Laboratory, School of Life Sciences, University of Sussex, Brighton BN1 9Q, UK
2 Pharmaceutical Engineering and 3D Printing (PharmE3D) Lab, Division of Molecular Pharmaceutics and Drug Delivery, College of Pharmacy | University of Texas at Austin, Austin, Texas 78712, USA
* Corresponding authors: M. Maniruzzaman (M.Maniruzzaman@austin.utexas.edu, M.Maniruzzaman12@gmail.com) and A. Nokhodchi (a.nokhodchi@sussex.ac.uk)

Supplementary Data:

Supp. Table 1. FDM printability and quality of all four theophylline-loaded filaments.

Filament of Formulation No.	Quality of Printing
F1	Printable & Good Quality
F2	Printable & Good Quality
F3	Printable, Acceptable Quality
F4	Printable, Poor Surface Finish
